# Neural Invasion in Pancreatic Cancer: The Past, Present and Future

**DOI:** 10.3390/cancers2031513

**Published:** 2010-07-14

**Authors:** Ihsan Ekin Demir, Güralp O. Ceyhan, Florian Liebl, Jan G. D’Haese, Matthias Maak, Helmut Friess

**Affiliations:** Department of Surgery, Klinikum rechts der Isar, Technische Universität München, Munich, Germany; E-Mails: demir@chir.med.tu-muenchen.de (I.E.D.); ceyhan@chir.med.tu-muenchen.de (G.O.C.); liebl@chir.med.tu-muenchen.de (F.L.); dhaese@chir.med.tu-muenchen.de (J.G.D.); maak@chir.med.tu-muenchen.de (M.M.)

**Keywords:** neural invasion, neurotrophic factors, pancreatic neuropathy, neuroplasticity, neurotropism

## Abstract

In the past 15 years, invasion of nerves by cancer cells has escaped from its role as a mere bystander in cancer biology and turned into an attractive niche to study the heterotypic interaction between cancer cells and neurons. Today, neural invasion (NI) in pancreatic cancer (PCa) stands out due to the recent demonstration of its association with tumor progression, local recurrence and neuropathic pain. Accordingly, recent research on NI in PCa revealed the critical involvement of numerous nerve- or cancer cell-derived molecules in several novel *in vitro* and *in vivo* models of NI, which, however, still need further major improvement.

## 1. Introduction

In the “Synthesis” section of their monumental article from 2000 entitled “The hallmarks of cancer”, Douglas Hanahan and Robert Weinberg pointed out what may be the most important factor for the correct understanding of the biology of cancer: They complained that current experimental models of cancer neglected the crucial dependence of cancer formation “upon changes in the heterotypic interactions between incipient tumor cells and their normal neighbors” [1]. They added that “continuing elucidation of cancer pathogenesis will depend increasingly upon heterotypic organ culture systems *in vitro* and evermore refined mouse models *in vivo*” [1]. Accordingly, cancer research in the past 10 years has not only produced a gigantic amount of scientific information on the intrinsic, genetic alterations in cancer cells, but also witnessed a dramatic increase in the number of efforts to understand the impact of tumor microenvironment on cancer formation and progression. As a result of these efforts, one of the “neighbors” of cancer cells that moved into focus was the nervous system in the organ of cancer origin. Indeed, in several human cancers, nerves have long been observed to be frequently invaded by cancer cells [2,3,4,5]. However, the human malignancy in which the invasion of nerves by cancer cells has attracted by far the highest degree of academic attention and research activity has been pancreatic cancer (PCa) [6,7,8]. Despite increasing apprehension of its pathomechanism, the prognosis of PCa has not undergone any significant improvement in the past 50 years: PCa still allows a median survival time of a mere six to eight months after diagnosis, and the five-year overall survival rate remains around 5% [9,10,11]. In addition to this utmost dismal prognosis, what motivated researchers to increasingly investigate neural invasion (NI) was the high prevalence of NI in PCa, reaching up to 100% [6,8], and its strong association with local recurrence after curative tumor resection and thus worsened prognosis [12,13,14]. Initial research on NI in PCa concentrated on its detailed anatomic and histopathological characterization. However, over time, research on NI evolved to be a fascinating niche to study the complex interaction between cancer cells and nerves at the molecular level. At the same time, the resulting emergence of novel *in vitro* and *in vivo* models is progressing at a hitherto unrivalled pace. Therefore, the present review aims at illustrating the historical evolution of research on NI in PCa into the present time and underlining potential future directions that might lead to an even better understanding of this peculiar pathologic phenomenon in PCa. 

## 2. Historical Evolution of Research on Neural Invasion in Pancreatic Cancer

Anatomists and pathologists seem to have recognized neural cancer cell invasion as early as the beginning of 19th century [15]. Interestingly, the first attempts to provide a pathomechanistic explanation for its occurrence date back to 1905, when Ernst attributed perineural invasion to the growth of chondrosarcoma cells within perineural lymphatic vessels [16]. It was again around the beginning of the 1930s when the other major theory, *i.e.*, “neurotropism”, emerged as an alternative explanation for the generation of NI [17]. In this regard, Jentzer was the first one to note that cancer cells made use of nerves as an additional path for invasion. According to Jentzer, this process should not be regarded as a mere invasion of nerves by cancer cells because cancer cells actually exploited nerves to reach distant destinations during their spread. Thereby, Jentzen underlined that this phenomenon should not be termed “neurophilia” but rather “neurotropism” [17]. As later studies failed to demonstrate the presence of perineural lymphatic vessels [18], the theory of neurotropism persisted into the present time as the most widely accepted explanation for the occurrence of NI in human cancers, including PCa. 

What may be regarded as a lighthouse for increased understanding of NI, particularly in PCa, was the pioneering study by Dale Bockman. By examining invaded nerves in the pancreas by electron microscopy, he found that cancer cells are by no means limited to the perineurium but delve deeper into the endoneurium and establish a close spatial association with endoneural structures including axons and Schwann cells [19]. Moreover, one of the major characteristics of NI noted by Bockman was “neural damage”: the perineurium was disrupted, the integrity of the whole nerve was distorted, and axons had a severely edematous appearance as a result of the invasion by PCa cells [19]. Aside from providing the most detailed ultrastructural analysis of NI in PCa, Bockman postulated a key role for the transforming growth factor alpha (TGF-α) in this process: invaded nerves demonstrated high immunoreactivity for the TGF-α, whereas PCa cells expressed high levels of epidermal growth factor receptor (EGFR), the natural receptor for TGF-α. Hence, nerves in PCa could represent a major source of growth factors for PCa cells, making nerves attractive targets of invasion for PCa cells. This notion of nerve-based growth support for PCa cells has dominated into the present time, paving the path for numerous studies that recently identified similar growth factors potentially originating from nerves during the process of NI, as explained later in this article in detail.

But is neurotropism sufficient to induce neural invasion? In other words, why have some cancers like PCa consistently been identified to bear high degrees of NI as opposed to others? A simple but fundamental option to answer these questions was put forward by several authors from Japan based on the normal anatomic localization of the human pancreas: The pancreas as a retroperitoneal organ is surrounded by several types of neural plexus at its dorsal aspect, including the celiac plexus, dorsal hepatic plexus and the plexus around the superior mesenteric artery. Extensive and detailed cadaver studies from Japan demonstrated that extrapancreatic neural plexus invasion is encountered in around 80% of cases and shows a partially repetitive pattern depending on the localization of the pancreatic tumor: tumors of the pancreatic head tend to grow towards the celiac plexus and ganglion and along the posterior hepatic plexus whereas the pancreatic body and tail tumors spread to the splenic plexus, celiac plexus and ganglion [20,21,22]. It has also been demonstrated that this pattern of spread follows the peripancreatic vasculature: Patients with tumors in the ventral pancreas frequently had pancreatic head plexus invasion and invasion along mesenteric arterial plexus, whereas patients with dorsal tumors had invasion into plexus around common hepatic artery and portal vein [22,23]. These results from cadaver studies have been largely confirmed by very recent imaging studies that employed contrast-enhanced multi-detector row computer tomography (MDCT) [23,24]. Based on these observations, Japanese authors have advocated extensive radical surgery involving dissection of the peri- and retropancreatic plexus to improve survival [7,20,21]. While some studies proposed better survival rates for patients undergoing extended resection [25,26], further studies demonstrated neither a reduction in local recurrence rates nor improved survival as a result of this procedure [27,28]. Therefore, the overall prognostic impact of such a radical resection still remains to be determined in larger cohorts. 

However, what may be considered a “twist of fate” in research on NI in PCa was the recognition of the potential role of NI in the generation of *pain* in PCa patients. While Bockman also favored further investigation of NI due its potential association with pain in PCa [19], it was the cutting-edge study by Zhu *et al.* which for the first time demonstrated a robust correlation between the intrapancreatic expression of the nerve growth factor (NGF), the frequency of perineural invasion and the degree of pain sensation in PCa patients [29]. This key correlation could be verified by two further studies that demonstrated increased abdominal or back pain in patients with increased intrapancreatic expression of NGF and its receptor TrkA [30,31]. These studies thereby not only added a novel aspect to the nociceptive properties of NGF by attributing it a nociceptive role in a “visceral” organ, but also paved the path for further investigation of nerve-derived factors in the pathomechanism of PCa. The consideration and intensified investigation of such neural molecules in the pathomechanism of three inter-linked phenomena, *i.e.*, perineural invasion, pain and PCa progression, inaugurated the present episode of research on NI in PCa. 

## 3. Current Concepts on Neural Invasion in Pancreatic Cancer

The disaffiliation of NI from the role of a mere bystander in PCa and the increasing recognition of its importance fostered the motivation to achieve an even more detailed characterization of its natural and histopathological properties. As a result, two studies in current medical literature emerge as the most comprehensive ones, describing multiple aspects of NI in PCa:

First, in the study by Kayahara *et al.*, the investigators examined consecutive sections of surgically resected PCa specimens to investigate the mechanism and mode of spread in NI. They could thereby identify five main patterns of cancer cell growth along nerves: (1) direct invasion of the nerves; (2) continuous tumor cells growth in the perineural space; (3) branching of the growing tumor mass along neural branches; (4) formation of a foremost growth cone of tumor cells; and (5) direct invasion of contiguous lymph nodes. This study by Kayarara *et al.* bears the importance of being the first study which tried to identify an anatomical mechanism for the manifestation of NI and particularly extrapancreatic neural plexus invasion. Therefore, it demonstrated that NI involves continuous growth of PCa cells along nerves towards the extrapancreatic neural plexus. However, it should be noted the prevalence of continuous cancer cell growth along nerves should not necessarily be considered as the only mechanism for spread of NI in PCa. Interestingly, it could be demonstrated that in nearly more than 50% of all examined PCa specimens, NI can be encountered in normal pancreatic areas that are distant from the main tumor, a phenomenon which was termed “nex” [32,33]. Moreover, the presence of nex strongly correlated with the presence of extrapancreatic nerve plexus invasion, and thus should be regarded as a poor prognostic sign [32,33]. 

**Figure 1:**
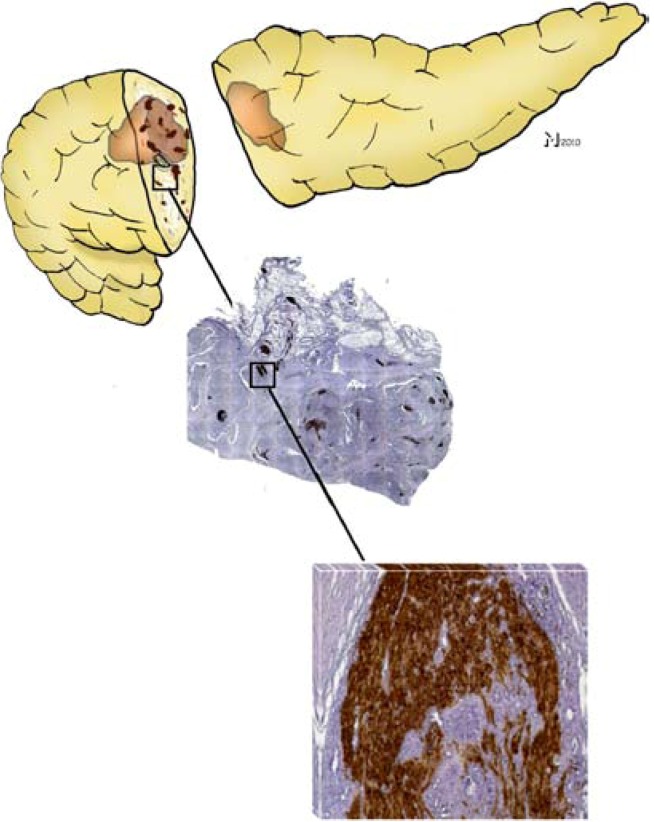
Neural invasion (NI) in pancreatic cancer (PCa) and pancreatic neuroplasticity are interlinked phenomena. NI is encountered in nearly all cases of ductal adenocarcinoma of the pancreas either within or adjacent to the main tumor mass (top panel). Histopathologic examination of the pancreatic tumor together with peripancreatic areas demonstrates a drastically increased number and size of intrapancreatic nerves (middle panel, 25× magnified, nerves were immunohistochemically/brown-labeled with the pan-neuronal marker protein gene product 9.5/PGP9.5). A closer look at these hypertrophic nerves reveals a high frequency of NI, even in the endoneural space (lower panel; 200x magnification, cancer cells correspond to the hematoxylin-counterstained areas within the brown-PGP9.5-immunostained nerve).

**Figure 2:**
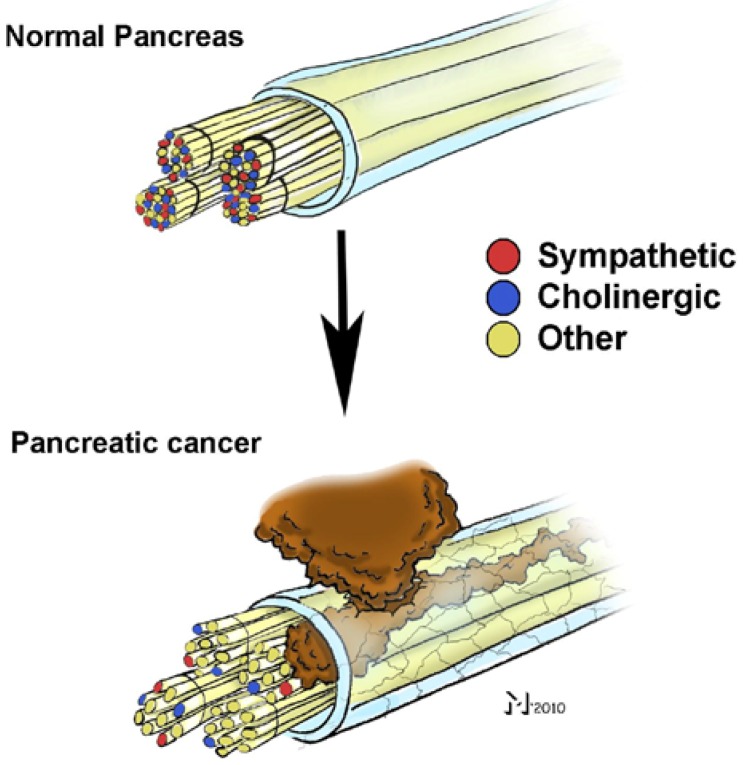
Neural invasion (NI) in pancreatic cancer (PCa) is associated with a “remodeling” of intrapancreatic nerves. Analysis of nerve fiber qualities in the hypertrophic, sprouting and invaded nerves in PCa revealed that the proportion of sympathetic and cholinergic nerve fibers in these nerves is much smaller than in normal pancreatic nerves. Therefore, the quality of the remaining fibers in these nerves remains to be identified.

In the second study, our group aimed at performing the currently largest-scale systematic analysis of NI in PCa by including a total of 546 patients with different pancreatic tumors, *i.e*., ductal adenocarcinoma, neuroendocrine tumors, benign pancreatic tumors, *etc*. [34]. One of the first findings of that study was the demonstration of its multiple manifestations: as initially described by Bockman, cancer cells were seen to be not confined to the perineural layer, but to penetrate into the endoneurium and towards nerve fibers. In order to attain a proper quantification of its different manifestations, we introduced a novel standardized scoring system that differentiates between perineural (PNI) and endoneural invasion (ENI). The mentioned study demonstrated that ductal adenocarcinoma of the pancreas bears a far higher degree of NI in comparison to all other pancreatic tumors (Figure 1). Furthermore, this study could also strengthen the previously suggested involvement of NI in pain sensation: patients whose nerves exhibited ENI had more frequent and intense pain than patients who merely had PNI. Moreover, PCa was seen to be not only characterized by NI, but also by several other neuroplastic alterations including neural hypertrophy, increased neural density and neural inflammatory cell infiltration (“pancreatic neuritis”) (Figure 1) [34]. Strikingly, the same study also identified a prominent association between the severity of NI in PCa and the extent of intrapancreatic neuroplastic alterations: Enlarged and sprouting nerves were at the same time the ones that exhibited NI. In a subsequent study, we could also demonstrate that these plastic alterations additionally included a remarkable switch in the pancreatic innervation quality: nerves invaded by cancer cells harbored a much lower amount of sympathetic and cholinergic nerve fibers (Figure 2) [35]. There is increasing evidence that these neuroplastic alterations in PCa are due to the neurotrophic attributes of the microenvironment in PCa, as demonstrated by another recent study by our group. In this study, we observed that tissue extracts of PCa, PCa cell supernatants and supernatants of human pancreatic stellate cells as main generators of desmoplasia can all induce axonal sprouting, increased neurite density and perikaryonal hypertrophy of neurons under *in vitro* conditions [36]. 

The strong association between pancreatic neuroplastic alterations and pain sensation by PCa patients points to the presence of a “neuropathic” pain mechanism in PCa, since the currently accepted definition for neuropathic pain is “pain arising as a direct consequence of a lesion or disease affecting the somatosensory system” [37]. Obviously, the neural damage in conjunction with NI plays a major role in this visceral neuropathy. Hence, the introduction of neuropathic analgesic regimens into the treatment of patients with advanced PCa may be of potential benefit.

## 4. Molecular Mediators of Neural Invasion in Pancreatic Cancer: the Emergence of Novel *in Vitro* Models

The past 10 years of NI research has also been marked by a constant rise in the number of studies that aimed at identifying potential mediators of the attraction between PCa cells and nerves. It is quite remarkable that these efforts have recently given birth to numerous novel advanced *in vitro* models of NI in PCa. In the first of these models, Dai *et al.* co-cultivated the human PCa cell line MiaPaCa-2 together with mouse dorsal root ganglia (DRG) and observed enhanced PCa cell growth than among non-co-cultured control PCa cells [38]. This enhanced growth was also evidenced by the over-expression of prosurvival genes like MALT1 and TRAF [38]. Importantly, the authors also noted reciprocity between neurite outgrowth and PCa cell proliferation in this assay: The better the cancer cells grew, the more prominent was the neurite outgrowth from DRG. 

In a recent study, our group presented another *in vitro* approach that allows a precise spatiotemporal monitoring of NI by PCa cells. There, we co-cultivated different PCa cell lines together with rat DRG or myenteric plexus (MP) cells in a three-dimensional (3D) extracellular matrix (ECM)-based migration assay. As opposed to all currently available *in vitro* models in the literature, the migration assay we presented requires an initial clear-cut physical separation of PCa cells and neurons, as it is the case under *in vivo* conditions. Owing to such an initial physical separation, the model allows the exact demonstration of the initial reaction of each cell type to the co-culture conditions. Furthermore, the model includes a pre-defined migration path for PCa cells, *i.e.*, defined ECM-bridges, which ensures the formation of a signal molecule gradient for any chemotactic factor. This way, we could demonstrate that PCa cells facing the neurons react to the presence of neurons with a typical morphological alteration, *i.e.*, flattening, grouping, colony formation and spike-like cellular polarization directed towards neurites. Moreover, PCa cells progressively come into contact with neurites along which they migrate in a targeted fashion. As suggested by Dai *et al.*, the co-presence of PCa cells and neurons was seen to be marked by a mutual growth support and tropism. Furthermore, one of the major strengths of the presented model was the inclusion of MP cells of the enteric nervous system (ENS). It should be considered that the extrinsic component of the pancreatic innervation, *i.e.*, nerve fibers running within the vagal and splanchnic nerves, originate from DRGs or vagal nuclei, respectively, whereas the intrinsic component is represented by pancreatic neurons derived from enteric neurons [39,40]. Therefore, the usage of MP and DRG cells provides a much more realistic model for studying the whole pancreatic innervation than using the DRG alone [6]. In the same study, we also monitored the quantitative alterations in the expression of neurotrophins, their receptors and the members of the glial-cell-derived neurotrophic factor (GDNF) family. Interestingly, while the majority of these factors did not demonstrate major expression changes, the nerve growth factor (NGF) increased steadily throughout the migration process of 120 hours [6]. Hence, the presented 3D model on the one hand confirmed the previously suggested crucial role of NGF in the NI [29], but also stood out as a promising model to identify further key mediators of NI in future studies.

**Figure 3:**
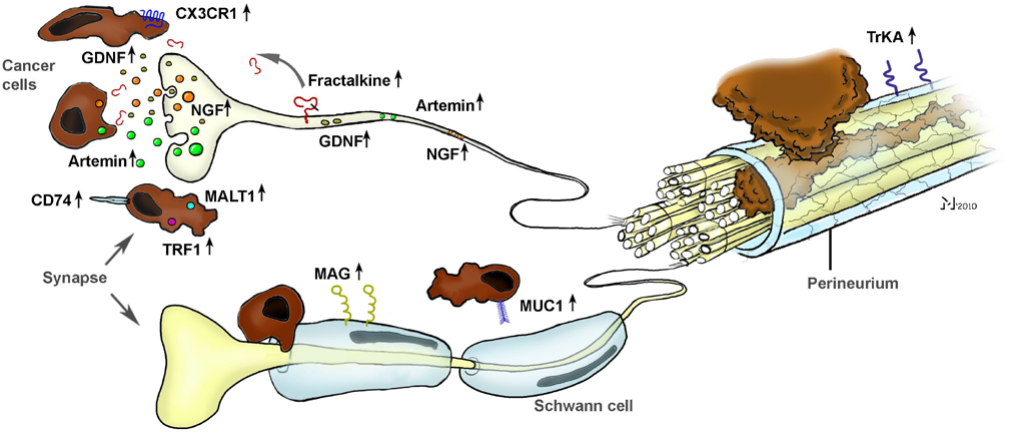
The interplay between neuronal and cancer-cell-derived factors in the generation of neural invasion (NI) in pancreatic cancer (PCa). Research from the past 10 years has revealed a crucial involvement of several neurotrophic factors (e.g., NGF, GDNF, artemin), chemokines (e.g., fractalkine), pro-survival genes (MALT1, TRAF) and cell-surface receptors (CD74, MAG) in the neuro-cancer interactions and NI in PCa. Please see the manuscript for the respective references.

As evidenced by other studies, NGF seems to be only one of several other molecular actors in the generation of NI (Figure 3). The potential role of chemokines was quickly recognized and investigated in a recent study by Marchesi *et al.*, who aimed at unveiling the involvement of the neuronal chemokine fractalkine (CX3CL1) in NI [41]. They could show that the neural immunoreactivity for the receptor of fractalkine, *i.e.*, CX3CR1, was significantly higher in perineural invasive lesions of PCa, and it was the CX3CR1-transfected, *in vivo* implanted PCa cells that exhibited a remarkable infiltration of peripheral nerves (Figure 3). Overall, they could prove that the CX3CR1 receptor may be involved in PCa neurotropism and serve as an independent risk factor for early local recurrence. Therefore, the CX3CR1-CX3CL1 axis was pointed out as a valuable therapeutic target to prevent tumor recurrence and progression in PCa [41]. Further studies shedding light on the role of chemokines in NI are underway.

In harmony with these studies, nerve-derived molecules have recently been the focus of further studies on NI (Figure 3). From these, Hibi *et al.* could show that the expression of synuclein gamma, which is involved in several neurodegenerative diseases, is a predictor of overall survival. Furthermore, in mouse perineural invasion and orthotopic transplantation models, stable synuclein-γ suppression by short hairpin RNA significantly reduced the incidence of perineural invasion and liver and lymph node metastasis [42]. A similarly impaired survival and increased rates of perineural invasion were also demonstrated for the intrapancreatic over-expression of neurite growth-promoting factor pleiotrophin [43]. Furthermore, Gil *et al.* demonstrated in a recent *in vitro* model involving DRG and PCa cell lines a major role for GDNF in the neurite-targeted migration of PCa cells: DRG derived from mice deficient in GDNF failed to attract PCa cells, and systemic blockade of the receptor tyrosine kinase RET, which is a downstream target of GNDF, could prevent NI toward the spinal cord and paralysis in mice [44]. A similar role for another GDNF family member, namely artemin, could be shown by our group, as artemin significantly increased the invasiveness of PCa cells and was over-expressed in PCa tissue [12].

As part of recent efforts to obtain a more comprehensive analysis and overview of the potential molecular actors in NI, Abiatari *et al.* recently reported on the generation of a transcriptome signature of NI in PCa by means of another *in vitro/ ex vivo* model. In their study, they co-cultivated human PCa cell lines with explanted rat vagus nerves and analyzed the consensus set of genes differentially regulated in all highly *versus* less nerve-invasive PCa cells [45]. Thereby, they could identify the differential expression of kinesin family member 14 (KIF14) and Rho-GDP dissociation inhibitor beta (ARHGDIbeta) in perineural invasion, which was confirmed on the RNA and protein levels in human PCa specimens. Moreover, small interfering RNA-mediated downregulation of KIF14 and ARHGDIbeta resulted in diminished perineural invasion [45]. Based upon this transciptome analysis, the authors put forward two other molecules, *i.e.*, the microtubule-associated protein MAPRE2 and the nuclear protein YPEL2, as molecules with derangements in their expression during NI in PCa [46,47].

In another recent outstanding study, Swanson *et al.* provided a novel insight to the neuro-cancer interactions by identifying the receptor for the transmembrane mucin MUC1 in the pancreas [48]. MUC1 is overexpressed and differentially glycosylated in several adenocarcinomas including PCa, and this aberrant glycosylation is associated with monosialyl and disialyl T-structure [48]. They could show that MUC1 expressed by cancer cells can bind to myelin-associated glycoprotein (MAG) expressed on Schwann cells of peripheral nerves, as evidenced by *in vitro* adhesion assays and immunohistochemical analysis of NI lesions in PCa specimens [49]. Hence, they could not only identify a novel heterotypic interaction, but provided a direct correlate of the close spatial relationship of nerves and cancer cells in PCa, as initially observed by Bockman [19]. 

## 5. *In Vivo* Models of Neural Invasion in Pancreatic Cancer: The Long Way to Perfection

The recent but steady increase in the number of *in vitro* models of NI in PCa seems to be in part paralleled by a similar tendency to generate realistic *in vivo* models. The first one of these *in vivo* approaches seems to have been performed by Eibl *et al.*, who attempted to simulate the high rates of local recurrence and perineural invasion after curative pancreatic tumor resection [50]. There, the investigators first performed an orthotopic implantation of the PCa cell lines MiaPaCa-2 (undifferentiated) and Capan-2 (well-differentiated) in nude mice pancreas and attempted a complete surgical resection of the tumor at four, six, and eight weeks post implantation. Nude mice who had received MiaPaCa-2 cells initially developed local tumor recurrence with extensive retroperitoneal nerve invasion and distant organ metastasis six weeks post implantation [50]. Hence, the authors achieved a realistic simulation of the local recurrence and NI associated with PCa in a murine model, which may well allow the study of the course of NI following tumor resection [50]. However, the main limitation of the presented model is that invasion of extrapancreatic neural plexus is a rather late event in PCa, and probably even early stage PCa localized to the pancreas already bears a prominent degree of intrapancreatic NI [7,19,32,33,34]. Therefore, the initial, and probably deciding, events leading to NI are unlikely to be studied by means of this model.

A frequently cited model of NI in PCa was created by Koide *et al.*: In their study, the investigators subcutaneously (s.c.) implanted seven different PCa cell lines with or without human peripheral nerves in non-obese diabetes/severe combined immunodeficient mice and analyzed the frequency of NI by these different cell lines [51]. Additionally, they performed an oligonucleotide microarray to obtain the expression profiles of high and low perineurally invasive cell lines. Four out of the seven selected cell lines presented invasion of the implanted human nerves; however, this invasion was very frequently accompanied by perineural inflammation. Therefore, the investigators refrained from further investigating the invasion of implanted peripheral nerves but limited themselves to the perineural invasion around mouse s.c. nerves. It was only two well-differentiated cell lines (Capan-1 and Capan-2) that invaded mouse s.c. nerves. Furthermore, they identified over-expression of CD74 in the perineurally invasive cells, which was confirmed by analysis of PCa tissue specimens. Altogether, the authors presented a possibility of investigating the process of NI in another xenograft model which is still limited by the preferential invasion by well-differentiated cell lines and the major lack of concomitant neuroplastic alterations that are idiosyncratic for human PCa [34]. Moreover, the model does not involve the natural nerves of the actual end-organ, *i.e.*, pancreatic nerves, which are mainly autonomic nerves as opposed to the somatic subcutaneous nerves.

There is currently a single *in vivo* model of NI in PCa, which was designed with a primarily therapeutic intention. In this study, Gil *et al.* injected PCa cell lines into the perineurium of the sciatic nerve of athymic mice and monitored limb function for nine days after injection [52]. Strikingly, they could show that a single injection of an attenuated, replication-competent, oncolytic herpes simplex virus seven days after intraneural PCa cell injection can effectively treat invaded nerves without compromising physiological nerve function [52]. In a subsequent study, the same authors presented the utility of this model for intraoperative diagnosis of NI [53]. Specifically, after intrasciatic implantation of PCa cell lines, they injected the same attenuated virus, this time with enhanced green fluorescent protein expression (eGFP), into the sciatic nerve. By employing intraoperative fluorescent stereoscopic imaging to detect the eGFP, they could identify the nerves invaded by PCa cells [53]. Therefore, the authors advocated oncolytic viruses for enhanced diagnosis of PCa due to facilitated detection of invaded nerves that would be subject to resection, and as a novel therapy regimen. Undoubtedly, the efforts of Gil *et al.* occupy a special place due to the concomitant dedication of the group to generate novel therapeutic approaches to NI and to elucidate its exact pathomechanism. However, the actual sensitivity of their method to fully detect and to eradicate NI remains to be determined.

## 6. Future Directions in Research on Neural Invasion in Pancreatic Cancer: “The Best Is Yet to Come”

The breathtaking evolution of research on NI and the rapidly growing scientific interest in NI are heralding an era of numerous further discoveries in the neuro-cancer interactions in PCa. The above outlined specifics of NI research at the same time point out the multiple aspects of NI that are waiting to be unveiled. Certainly, the achievement of future discoveries in this field is going to depend on the careful and critical appraisal of current findings and the generation of even more interesting questions pertinent to the pathogenesis of NI. 

To name some of potentially interesting aspects, we are convinced that the main hypothesis for the generation of NI, *i.e.*, the neurotropism of PCa cells, has to be carefully reviewed. As evidenced by previous studies, holding cancer cells responsible for every aspect of NI and omitting the contribution of neurons to this process may turn out to be totally incorrect. The role of NGF, GDNF, artemin, fractalkine and CX3CR1, MAG and several other nerve-derived molecules should serve as a motivation for comprehensive screening of alterations in the expression of neuronal molecules during NI. 

Second, despite the impressive and rapid development of novel astute *in vitro* models, all of the NI models need to be verified for their specificity for PCa and NI in general. This can be achieved via inclusion of, e.g., cancer cells that normally exhibit little or no NI, or via co-culturing of cancer cells with non-neuronal cells. What is more, although several *in vitro* models make use of extracellular matrix (ECM) gel-based assays, the intrinsic ability of ECM components like fibronectin, laminin and collagen subtypes to guide cellular migration should not be underestimated. As shown in a recent study, these molecules can be preferentially expressed by the perineurium and guide cancer cells in their migration along nerves [54]. Therefore, for studies primarily investigating chemotactic agents in NI, the exclusion of large amounts of ECM proteins may help with the elimination of confounding factors. 

Finally, there still seems to be a need for improved *in vivo* models of NI in PCa since current models fail to represent the NI in a realistic manner due to the above mentioned reasons. Considering these, an ideal *in vivo* model of NI in PCa could be characterized by (1) origination of the tumor from the pancreas; (2) the presence of a ductal adenocarcinoma in the pancreas; (3) the presence of NI even at early stages of PCa; (4) the specific extension of NI towards the extrapancreatic neural plexus; (5) no need for iatrogenic injection of tumor cells through the epineurium; (6) accompanying neuropathic and desmoplastic alterations and (7) concurrent pain. An intriguing question in this regard is whether these features can be realized in murine models of NI due to the normal anatomic localization of the pancreas in these animals when compared to the retroperitoneal localization of human pancreas. The human pancreas is surrounded by numerous neural plexus at its rear border [20,21,22]; however, similar studies on retropancreatic neural plexus in mice or rats are not present. Therefore, careful investigation of the peripancreatic plexus anatomy may help better predict the usefulness of animal models to simulate human NI in PCa.

## 7. Conclusions

Although more than 100 years old, research on the invasion of nerves by pancreatic cancer cells has only gained speed in the past 10 years, owing to the recognition of the close relationship between NI, pain, survival and tumor recurrence. Accordingly, the current expectations associated with research on NI comprise discovery of novel tools to limit local tumor spread, recurrence and prevention or treatment of neuropathic pain due to NI. Undoubtedly, the fulfillment of these expectations necessitates proper and in-depth understanding of the multiple interactions between cancer cells and nerves. Invasion of nerves by PCa cells is traditionally attributed to a neurotropism of PCa cells, whereas recent findings underscore the reciprocity of the tropism between cancer cells and neurons. Despite the discovery of numerous molecular mediators of this tropism, most studies include genome-wide analyses carried out in the cancer cells only, possibly due to the technical difficulties associated with the performance of similar studies in neuronal cells. However, as demonstrated by recent advanced in *in vitro* and *in vivo* models, the functional modulation of such neuronal agents bears high diagnostic and therapeutic potential. Also in this fascinating field of research, future developments and discoveries heavily depend on the critical appraisal and refinement of the present experimental models, as suggested by Hanahan and Weinberg at the end of the last millennium [1]. To conclude with their own words, it remains to be hoped that, for the generation of effective therapeutic regimens in PCa, the future research on NI in PCa is also going to assume a direction “where the complexities of the disease, described in the laboratory and clinic, will become understandable in terms of a small number of underlying principles” [1].

## References

[1] Hanahan D., Weinberg R.A. (2000). The hallmarks of cancer. Cell.

[2] Bhuiya M.R., Nimura Y., Kamiya J., Kondo S., Fukata S., Hayakawa N., Shionoya S. (1992). Clinicopathologic studies on perineural invasion of bile duct carcinoma. Ann. Surg..

[3] Liebig C., Ayala G., Wilks J., Verstovsek G., Liu H., Agarwal N., Berger D.H., Albo D. (2009). Perineural invasion is an independent predictor of outcome in colorectal cancer. J. Clin. Oncol..

[4] Takubo K., Takai A., Yamashita K., Yoshimatsu N., Kitano M., Sasajima K., Fujita K. (1985). Light and electron microscopic studies of perineural invasion by esophageal carcinoma. J. Natl. Cancer Inst..

[5] Villers A., McNeal J.E., Redwine E.A., Freiha F.S., Stamey T.A. (1989). The role of perineural space invasion in the local spread of prostatic adenocarcinoma. J. Urol..

[6] Ceyhan G.O., Demir I.E., Altintas B., Rauch U., Thiel G., Muller M.W., Giese N.A., Friess H., Schafer K.H. (2008). Neural invasion in pancreatic cancer: A mutual tropism between neurons and cancer cells. Biochem. Biophys. Res. Commun..

[7] Kayahara M., Nakagawara H., Kitagawa H., Ohta T. (2007). The nature of neural invasion by pancreatic cancer. Pancreas.

[8] Liu B., Lu K.Y. (2002). Neural invasion in pancreatic carcinoma. Hepatobiliary Pancreat. Dis. Int..

[9] Jemal A., Siegel R., Ward E., Hao Y., Xu J., Thun M.J. (2009). Cancer statistics, 2009. CA Cancer J. Clin..

[10] Cameron J.L., Crist D.W., Sitzmann J.V., Hruban R.H., Boitnott J.K., Seidler A.J., Coleman J. (1991). Factors influencing survival after pancreaticoduodenectomy for pancreatic cancer. Am J Surg.

[11] Niederhuber J.E., Brennan M.F., Menck H.R. (1995). The national cancer data base report on pancreatic cancer. Cancer.

[12] Ceyhan G.O., Giese N.A., Erkan M., Kerscher A.G., Wente M.N., Giese T., Buchler M.W., Friess H. (2006). The neurotrophic factor artemin promotes pancreatic cancer invasion. Ann. Surg..

[13] Kameda K., Shimada H., Ishikawa T., Takimoto A., Momiyama N., Hasegawa S., Misuta K., Nakano A., Nagashima Y., Ichikawa Y. (1999). Expression of highly polysialylated neural cell adhesion molecule in pancreatic cancer neural invasive lesion. Cancer Lett..

[14] Nagai H., Kuroda A., Morioka Y. (1986). Lymphatic and local spread of t1 and t2 pancreatic cancer. A study of autopsy material. Ann. Surg..

[15] Cruveilheir J., Bailliere J.N. (1835). Maladie des nerfs. Anatomie Pathologique du Corps Humain.

[16] Ernst P. (1905). Über das wachstum und die verbreitung bösartiger geschwülste insbesondere des krebses in den lymphbahnen der nerven. Beitr. Pathol. Anat..

[17] Jentzer A. (1930). Neurotropisme des céllules cancereuses: Clinique et thérapeutique des cancers neurotropes. Schweiz. Med. Wochenschr..

[18] Larson D.L., Rodin A.E., Roberts D.K., O'Steen W.K., Rapperport A.S., Lewis S.R. (1966). Perineural lymphatics: Myth or fact. Am. J. Surg..

[19] Bockman D.E., Buchler M., Beger H.G. (1994). Interaction of pancreatic ductal carcinoma with nerves leads to nerve damage. Gastroenterology.

[20] Yi S.Q., Miwa K., Ohta T., Kayahara M., Kitagawa H., Tanaka A., Shimokawa T., Akita K., Tanaka S. (2003). Innervation of the pancreas from the perspective of perineural invasion of pancreatic cancer. Pancreas.

[21] Nakao A., Harada A., Nonami T., Kaneko T., Takagi H. (1996). Clinical significance of carcinoma invasion of the extrapancreatic nerve plexus in pancreatic cancer. Pancreas.

[22] Makino I., Kitagawa H., Ohta T., Nakagawara H., Tajima H., Ohnishi I., Takamura H., Tani T., Kayahara M. (2008). Nerve plexus invasion in pancreatic cancer: Spread patterns on histopathologic and embryological analyses. Pancreas.

[23] Deshmukh S.D., Willmann J.K., Jeffrey R.B. (2010). Pathways of extrapancreatic perineural invasion by pancreatic adenocarcinoma: Evaluation with 3d volume-rendered mdct imaging. AJR Am. J. Roentgenol..

[24] Mochizuki K., Gabata T., Kozaka K., Hattori Y., Zen Y., Kitagawa H., Kayahara M., Ohta T., Matsui O. (2010). Mdct findings of extrapancreatic nerve plexus invasion by pancreas head carcinoma: Correlation with en bloc pathological specimens and diagnostic accuracy. Eur. Radiol..

[25] Kayahara M., Nagakawa T., Ueno K., Ohta T., Tsukioka Y., Miyazaki I. (1995). Surgical strategy for carcinoma of the pancreas head area based on clinicopathologic analysis of nodal involvement and plexus invasion. Surgery.

[26] Nagakawa T., Nagamori M., Futakami F., Tsukioka Y., Kayahara M., Ohta T., Ueno K., Miyazaki I. (1996). Results of extensive surgery for pancreatic carcinoma. Cancer.

[27] Levy M.J., Topazian M., Keeney G., Clain J.E., Gleeson F., Rajan E., Wang K.K., Wiersema M.J., Farnell M., Chari S. (2006). Preoperative diagnosis of extrapancreatic neural invasion in pancreatic cancer. Clin. Gastroenterol. Hepatol..

[28] Nakao A., Kaneko T., Takeda S., Inoue S., Harada A., Nomoto S., Ekmel T., Yamashita K., Hatsuno T. (2001). The role of extended radical operation for pancreatic cancer. Hepatogastroenterology.

[29] Zhu Z., Friess H., diMola F.F., Zimmermann A., Graber H.U., Korc M., Buchler M.W. (1999). Nerve growth factor expression correlates with perineural invasion and pain in human pancreatic cancer. J. Clin. Oncol..

[30] Dang C., Zhang Y., Ma Q., Shimahara Y. (2006). Expression of nerve growth factor receptors is correlated with progression and prognosis of human pancreatic cancer. J. Gastroenterol. Hepatol..

[31] Zhang Y., Dang C., Ma Q., Shimahara Y. (2005). Expression of nerve growth factor receptors and their prognostic value in human pancreatic cancer. Oncol. Rep..

[32] Takahashi T., Ishikura H., Kato H., Tanabe T., Yoshiki T. (1992). Intra-pancreatic, extra-tumoral perineural invasion (nex). An indicator for the presence of retroperitoneal neural plexus invasion by pancreas carcinoma. Acta Pathol. Jpn..

[33] Takahashi T., Ishikura H., Motohara T., Okushiba S., Dohke M., Katoh H. (1997). Perineural invasion by ductal adenocarcinoma of the pancreas. J. Surg. Oncol..

[34] Ceyhan G.O., Bergmann F., Kadihasanoglu M., Altintas B., Demir I.E., Hinz U., Muller M.W., Giese T., Buchler M.W., Giese N.A., Friess H. (2009). Pancreatic neuropathy and neuropathic pain--a comprehensive pathomorphological study of 546 cases. Gastroenterology.

[35] Ceyhan G.O., Demir I.E., Rauch U., Bergmann F., Muller M.W., Buchler M.W., Friess H., Schafer K.H. (2009). Pancreatic neuropathy results in "Neural remodeling" And altered pancreatic innervation in chronic pancreatitis and pancreatic cancer. Am. J. Gastroenterol..

[36] Demir I.E., Ceyhan G.O., Rauch U., Altintas B., Klotz M., Muller M.W., Buchler M.W., Friess H., Schafer K.H. (2010). The microenvironment in chronic pancreatitis and pancreatic cancer induces neuronal plasticity. Neurogastroenterol. Motil..

[37] Treede R.D., Jensen T.S., Campbell J.N., Cruccu G., Dostrovsky J.O., Griffin J.W., Hansson P., Hughes R., Nurmikko T., Serra J. (2008). Neuropathic pain: Redefinition and a grading system for clinical and research purposes. Neurology.

[38] Dai H., Li R., Wheeler T., Ozen M., Ittmann M., Anderson M., Wang Y., Rowley D., Younes M., Ayala G.E. (2007). Enhanced survival in perineural invasion of pancreatic cancer: An *in vitro* approach. Hum. Pathol..

[39] Kirchgessner A.L., Adlersberg M.A., Gershon M.D. (1992). Colonization of the developing pancreas by neural precursors from the bowel. Dev. Dyn..

[40] Kirchgessner A.L., Gershon M.D. (1990). Innervation of the pancreas by neurons in the gut. J. Neurosci..

[41] Marchesi F., Piemonti L., Fedele G., Destro A., Roncalli M., Albarello L., Doglioni C., Anselmo A., Doni A., Bianchi P., Laghi L., Malesci A., Cervo L., Malosio M., Reni M., Zerbi A., Di Carlo V., Mantovani A., Allavena P. (2008). The chemokine receptor cx3cr1 is involved in the neural tropism and malignant behavior of pancreatic ductal adenocarcinoma. Cancer Res..

[42] Hibi T., Mori T., Fukuma M., Yamazaki K., Hashiguchi A., Yamada T., Tanabe M., Aiura K., Kawakami T., Ogiwara A., Kosuge T., Kitajima M., Kitagawa Y., Sakamoto M. (2009). Synuclein–gamma is closely involved in perineural invasion and distant metastasis in mouse models and is a novel prognostic factor in pancreatic cancer. Clin. Cancer Res..

[43] Yao J., Ma Q., Wang L., Zhang M. (2009). Pleiotrophin expression in human pancreatic cancer and its correlation with clinicopathological features, perineural invasion, and prognosis. Dig. Dis. Sci..

[44] Gil Z., Cavel O., Kelly K., Brader P., Rein A., Gao S.P., Carlson D.L., Shah J.P., Fong Y., Wong R.J. (2010). Paracrine regulation of pancreatic cancer cell invasion by peripheral nerves. J. Natl. Cancer Inst..

[45] Abiatari I., DeOliveira T., Kerkadze V., Schwager C., Esposito I., Giese N.A., Huber P., Bergman F., Abdollahi A., Friess H., Kleeff J. (2009). Consensus transcriptome signature of perineural invasion in pancreatic carcinoma. Mol. Cancer Ther..

[46] Abiatari I., Gillen S., DeOliveira T., Klose T., Bo K., Giese N.A., Friess H., Kleeff J. (2009). The microtubule–associated protein mapre2 is involved in perineural invasion of pancreatic cancer cells. Int. J. Oncol..

[47] Abiatari I., Kiladze M., Kerkadze V., Friess H., Kleeff J. (2009). Expression of ypel1 in pancreatic cancer cell lines and tissues. Georgian Med. News.

[48] Swanson B.J., McDermott K.M., Singh P.K., Eggers J.P., Crocker P.R., Hollingsworth M.A. (2007). Muc1 is a counter-receptor for myelin-associated glycoprotein (siglec-4a) and their interaction contributes to adhesion in pancreatic cancer perineural invasion. Cancer Res..

[49] Singh P.K., Wen Y., Swanson B.J., Shanmugam K., Kazlauskas A., Cerny R.L., Gendler S.J., Hollingsworth M.A. (2007). Platelet-derived growth factor receptor beta-mediated phosphorylation of muc1 enhances invasiveness in pancreatic adenocarcinoma cells. Cancer Res..

[50] Eibl G., Reber H.A. (2005). A xenograft nude mouse model for perineural invasion and recurrence in pancreatic cancer. Pancreas.

[51] Koide N., Yamada T., Shibata R., Mori T., Fukuma M., Yamazaki K., Aiura K., Shimazu M., Hirohashi S., Nimura Y., Sakamoto M. (2006). Establishment of perineural invasion models and analysis of gene expression revealed an invariant chain (cd74) as a possible molecule involved in perineural invasion in pancreatic cancer. Clin. Cancer Res..

[52] Gil Z., Rein A., Brader P., Li S., Shah J.P., Fong Y., Wong R.J. (2007). Nerve-sparing therapy with oncolytic herpes virus for cancers with neural invasion. Clin. Cancer Res..

[53] Gil Z., Kelly K.J., Brader P., Shah J.P., Fong Y., Wong R.J. (2008). Utility of a herpes oncolytic virus for the detection of neural invasion by cancer. Neoplasia.

[54] Ryschich E., Khamidjanov A., Kerkadze V., Buchler M.W., Zoller M., Schmidt J. (2009). Promotion of tumor cell migration by extracellular matrix proteins in human pancreatic cancer. Pancreas.

